# Overcoming noise in quantum teleportation with multipartite hybrid entanglement

**DOI:** 10.1126/sciadv.adj3435

**Published:** 2024-05-01

**Authors:** Zhao-Di Liu, Olli Siltanen, Tom Kuusela, Rui-Heng Miao, Chen-Xi Ning, Chuan-Feng Li, Guang-Can Guo, Jyrki Piilo

**Affiliations:** ^1^ CAS Key Laboratory of Quantum Information, University of Science and Technology of China, Hefei 230026, China.; ^2^ CAS Center For Excellence in Quantum Information and Quantum Physics, University of Science and Technology of China, Hefei 230026, China.; ^3^ Department of Physics and Astronomy, University of Turku, FI-20014 Turun yliopisto, Finland.; ^4^ Department of Mechanical and Materials Engineering, University of Turku, FI-20014 Turun yliopisto, Finland.; ^5^ Hefei National Laboratory, University of Science and Technology of China, Hefei 230088, China.

## Abstract

Quantum entanglement and decoherence are the two counterforces of many quantum technologies and protocols. For example, while quantum teleportation is fueled by a pair of maximally entangled resource qubits, it is vulnerable to decoherence. Here, we propose an efficient quantum teleportation protocol in the presence of pure decoherence and without entangled resource qubits entering the Bell-state measurement. Instead, we use multipartite hybrid entanglement between the auxiliary qubits and their local environments within the open–quantum system context. With a hybrid-entangled initial state, it is the decoherence that allows us to achieve high fidelities. We demonstrate our protocol in an all-optical experiment.

## Teaser

Noisy yet efficient quantum teleportation is demonstrated without entangled open-system qubits in Bell-state measurement.

## INTRODUCTION

Quantum entanglement manifests itself in correlations that span over arbitrarily long distances (*1*). Besides its great significance on the foundations of quantum mechanics, entanglement has found numerous applications in quantum information processing and quantum communication, e.g., quantum teleportation (*2*–*7*), superdense coding (*8*–*10*), and quantum key distribution (*11*–*13*). However, the unavoidable interactions between a quantum system and its environment can severely degrade the performance of these applications by means of decoherence (*14*). Avoiding any kind of decoherence is extremely demanding in practice, although many promising decoherence suppression protocols have been proposed. Some recent works have exploited delayed coherent quantum feedback (*15*–*17*), reservoir engineering with auxiliary subsystems (*18*–*20*), quantum error-correcting codes (*21*–*23*), dynamical decoupling (*24*–*26*), and decoherence-free subspaces (*27*, *28*).

Linear optics provides a particularly robust platform in which to perform different quantum information protocols and study the problematics with decoherence. Here, the system of interest often consists of the polarization degrees of freedom of individual photons, their frequency represents the environment, and the system-environment interaction is realized controllably in birefringent media (*3*, *7*, *9*, *27*–*32*). Although the total dynamics is unitary, the open system undergoes nonunitary dynamics, which is obtained by averaging over the environment. The resulting dephasing drives the coherence terms of a given system to zero while keeping its populations intact, corresponding to quantum-to-classical transition.

Here, we study quantum teleportation under dephasing in the afore-described linear optical framework. We attack dephasing by using multipartite hybrid entanglement. Hybrid entanglement means entanglement between different degrees of freedom (*33*, *34*), and it has been previously used in overcoming the probabilistic nature of discrete-variable teleportation (*35*) but not yet in our context. With hybrid entanglement, we can controllably scramble the open system’s phase information so that the subsequent dephasing later reassembles it instead of scrambling it even more. As a consequence, with dephasing appearing in the end of our teleportation protocol, we can transform system-environment correlations into coherences within the teleported state. Note that, throughout this article, we will use “decoherence” and “dephasing” irrespective of how the coherences evolve.

We achieve high fidelities without the resource qubits being entangled in the Bell-state measurement (BSM). Moreover, our protocol works without any frequency correlations. Although the environment in our case is controllable, our proof-of-concept work lays the groundwork for future technologies with the anticipation that as technology advances, similar control may be exerted over more realistic environmental factors.

## RESULTS

### Theoretical description

The standard quantum teleportation protocol goes as follows (*2*). Alice has a qubit whose unknown state ∣ϕ⟩ = α∣0⟩ + β∣1⟩ she wants to teleport to Bob. In view of this task, Alice and Bob have shared a Bell state. Alice performs a BSM on her pair of states, i.e., the one to be teleported and her part of the auxiliary state. The two become fully entangled, erasing the initial entanglement between Alice and Bob due to the monogamy of entanglement. Alice classically reports her result to Bob, who then applies a unitary on his state, matching with the reported Bell state. As a result, Bob’s state becomes ∣ϕ⟩.

**Fig. 1.**
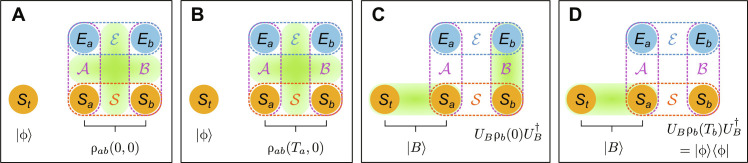
Stages of noisy quantum teleportation. ∣ϕ⟩ is the state to be teleported, initially carried by the system *S_t_*. *S*_*a*(*b*)_ is Alice’s (Bob’s) system/polarization, together forming the composite open system 𝒮 that is initially in the state ρ*_ab_*(0,0), whose nonlocality remains always hidden, i.e., while there is multipartite hybrid entanglement in the total system, the polarization degree of freedom alone does not display nonlocality. *E*_*a*(*b*)_ is Alice’s (Bob’s) environment/frequency, together forming the composite environment ℰ. 𝒜 (ℬ) is Alice’s (Bob’s) photon consisting of both the polarization and frequency degrees of freedom, and the green ovals represent entanglement. (**A**) Alice and Bob have shared the auxiliary state ∣Ψ(0,0)⟩. 𝒜 and ℬ are entangled through the initial probability amplitudes, while 𝒮 and ℰ are entangled through the phase functions θ*_j_*(*f_j_*). (**B**) Alice subjects her photon to dephasing noise, making the open system evolve from ρ*_ab_*(0,0) to ρ*_ab_*(*T_a_*,0), whose nonlocality is still hidden. (**C**) Alice performs BSM, entangling *S*_*t*_ and *S_a_*. At the same time, the hybrid entanglement transforms into system-environment entanglement on Bob’s side, i.e., between *S_b_* and *E_b_*. Alice classically communicates her result ∣*B*⟩ to Bob, who then, by operating with the matching unitary *U_B_*, obtains the polarization state *U_B_*ρ*_b_*(0)$U_{B}^{†}$ . (**D**) Bob subjects his photon to dephasing noise, which converts the *S_b_*-*E_b_* entanglement into coherences within *S_b_*, yielding ∣ϕ⟩.

The standard protocol assumes ideal conditions, i.e., no noise. We now consider a more realistic scenario, where the auxiliary state shared by Alice and Bob experiences local dephasing noise for the respective durations of *T_a_* and *T_b_*. The steps of our protocol are illustrated in Fig. 1.

Encoding the auxiliary qubits into the polarization degrees of freedom of two photons, having their frequencies act as local environments, and taking initial system-environment correlations into account, the total state of the two photons preceding the noise reads

$$\begin{array}{c} |\Psi (0,0)\rangle =\frac{1}{\sqrt{2}}\{|\;\mathrm{HV}\rangle \int df_{a}df_{b}g(f_{a},f_{b})e^{i[\theta_{\mathrm{aH}}(f_{a})+\theta_{\mathrm{bV}}(f_{b})]}\\ +|\mathrm{VH}\rangle \int df_{a}df_{b}g(f_{a},f_{b})e^{i[\theta_{\mathrm{aV}}(f_{a})+\theta_{\mathrm{bH}}(f_{b})]}\}|f_{a}f_{b}\rangle \end{array}$$ (1)

Here, *H* (*V*) denotes horizontal (vertical) polarization, *g*(*f_a_*, *f_b_*) is the probability amplitude corresponding to the joint frequency state ∣*f_a_f_b_*⟩ satisfying the normalization condition ∫*df_a_df_b_*∣*g*(*f_a_*, *f_b_*)∣^2^ = 1, and the θ-functions describe the initial polarization-frequency correlations. Having access to these functions, Alice and Bob can tailor the correlations to their liking. We make no other assumptions, e.g., about the frequency distribution.

The decoherence that the two polarization qubits will experience is described by the system-environment interaction Hamiltonian $H_{a}⊗l_{b}+l_{a}⊗{ℍ}_{b}$ , where the local Hamiltonians are of the pure-dephasing form (*14*)

$${ℍ}_{j}=-(n_{\mathrm{jH}}|H\rangle \left\langle H|+n_{\mathrm{jV}}|V\right\rangle \left\langle V|)⊗\int df_{j}2\pi f_{j}|f_{j}\right\rangle \langle \;f_{j}|$$ (2)

Here, *n*_*j*λ_ is the refractive index of a birefringent medium corresponding to Alice (*j* = *a*) or Bob’s (*j* = *b*) polarization component λ = *H*, *V*. This leads to the joint unitary evolution of the system and environment, *U_j_*(*t_j_*)∣λ⟩∣*f_j_*⟩ = *e*^*i*2π*fjnj*λ*tj*^∣λ⟩∣*f_j_*⟩, with *t_j_* denoting the interaction time. Because the open system and its environment together form a closed system, no information is truly lost regardless of initial correlations. This comes into play later. To take this kind of noise into account, Alice and Bob fix

$$\theta_{j}\left(f_{j}\right)=\theta_{\mathrm{jH}}\left(f_{j}\right)-\theta_{\mathrm{jV}}\left(f_{j}\right)=-2\pi f_{j}\Delta n_{j}T_{j}$$ (3)

independently of each other. Here, Δ*n_j_* = *n_jH_* − *n_jV_* denotes the birefringence.

Physically, imposing condition 3 on the state 1 entangles its (composite) polarization with the (composite) frequency, creating a multipartite hybrid-entangled state (*33*–*35*), and this can be done, e.g., with spatial light modulators [SLMs; (*32*)]. How this hybrid entanglement is distributed between Alice and Bob depends on the initial probability amplitudes. With our choices, for example, there is no entanglement in the open system or its environment alone, but rather the local system-environment states of Alice and Bob are entangled with each other (see the Supplementary Materials for more details).

Before having any noise, the bipartite polarization state, obtained by taking partial trace of ∣Ψ(0,0)⟩⟨Ψ(0,0)∣ over frequency, reads

$$\rho_{\mathrm{ab}}\left(0,0\right)=\frac{1}{2}[\begin{array}{cccc} 0 & 0 & 0 & 0\\ 0 & 1 & \Lambda_{ab}\left(0,0\right) & 0\\ 0 & \Lambda_{ab}^{*}\left(0,0\right) & 1 & 0\\ 0 & 0 & 0 & 0 \end{array}]$$ (4)

where Λ*_ab_*(*t_a_*, *t_b_*) = *df_a_df_b_*∣*g*(*f_a_*, *f_b_*)∣^2^ exp {*i*[θ*_a_*(*f_a_*) + 2π*f_a_Δn_a_t_a_* − θ*_b_*(*f_b_*) − 2π*f_b_Δn_b_t_b_*]} is the bivariate decoherence function governing the decoherence dynamics of the auxiliary state. With *T_j_* ≫ 0, we obtain the approximate form ρ*_ab_*(0, 0) ≈ (∣*HV*⟩⟨*HV*∣ + ∣*VH*⟩⟨*VH*∣)/2 that is (seemingly) local by its nature, i.e., its nonlocality is “hidden.”

Alice then removes her contribution of the polarization-frequency correlations described by θ*_a_*(*f_a_*) = −2π*f_a_*Δ*n_a_T_a_* by letting her auxiliary photon go through a birefringent crystal with the birefringence Δ*n_a_* and length *cT_a_*. It should be stressed that it need not be Alice who operates the SLM and implements noise. It might as well be a third party that prepares the auxiliary photons and sends one to Alice via noisy channel.

Because *T_b_* ≫ 0, we still have the decoherence function Λ(*T_a_*, 0) ≈ 0 and, therefore, the mixed state ρ*_ab_*(*T_a_*, 0) ≈ (∣*HV*⟩⟨*HV*∣ + ∣*VH*⟩⟨*VH*∣)/2. On the other hand, denoting the state being teleported by ∣ϕ⟩ = α∣*H*⟩ + β∣*V*⟩,the total state of the three photons is

$$|\Omega \left(T_{a},0\right)\rangle =\frac{1}{\sqrt{2}}|\phi \rangle \left[|\mathrm{HV}\rangle |\xi_{\mathrm{HV}}\left(T_{a},0\right)\rangle +|\mathrm{VH}\rangle |\xi_{\mathrm{VH}}\left(T_{a},0\right)\rangle \right]$$ (5)

where ∣ξ_λλ′_(*T_a_*,0)⟩ = ∫ *df_a_df_b_g*(*f_a_*, *f_b_*) exp {*i*[θ_*a*λ_(*f_a_*) + 2π*f_a_n*_*a*λ_*T_a_* + θ_*b*λ′_(*f_b_*)]}∣*f_a_f_b_*⟩. Note that, in Eq. 5, we have written the state to be teleported first, then Alice’s auxiliary qubit, and lastly Bob’s auxiliary qubit and that we will keep this order in the following. Namely, Eq. 5 can also be written in the form

$$\begin{array}{cc} |\Omega (T_{a},0)\rangle & =\frac{1}{2}|\Phi^{+}\rangle \left[\beta |H\rangle |\xi_{\mathrm{VH}}\left(T_{a},0\right)\rangle +\alpha |V\rangle |\xi_{\mathrm{HV}}\left(T_{a},0\right)\rangle \right]\\ & +\frac{1}{2}|\Phi^{-}\rangle \left[-\beta |H\rangle |\xi_{\mathrm{VH}}\left(T_{a},0\right)\rangle +\alpha |V\rangle |\xi_{\mathrm{HV}}\left(T_{a},0\right)\rangle \right]\\ & +\frac{1}{2}|\Psi^{+}\rangle \left[\alpha |H\rangle |\xi_{\mathrm{VH}}\left(T_{a},0\right)\rangle +\beta |V\rangle |\xi_{\mathrm{HV}}\left(T_{a},0\right)\rangle \right]\\ & +\frac{1}{2}|\Psi^{-}\rangle \left[\alpha |H\rangle |\xi_{\mathrm{VH}}\left(T_{a},0\right)\rangle -\beta |V\rangle |\xi_{\mathrm{HV}}\left(T_{a},0\right)\rangle \right] \end{array}$$ (6)

where ∣Φ^±^⟩ = (∣*HH*⟩ ± ∣*VV*⟩)/√2 and ∣Ψ^±^⟩ = (∣*HV*⟩ ± ∣*VH*⟩)/√2 are the Bell states.

Alice performs BSM on her pair of qubits. This simultaneously entangles her polarization qubits and remotely transforms the hybrid entanglement into local system-environment entanglement on Bob’s side. Alice communicates her result ∣*B*⟩ to Bob, who then applies a matching unitary operation *U_B_* on his qubit ρ*_b_*(0). The matching unitaries are

$$U_{B}=\left\{ \begin{array}{c} \sigma_{x}\;\text{for}\;|\Phi^{+}\rangle \\ i\sigma_{y}\;\text{for}\;|\Phi^{-}\rangle \\ l\;\text{for}\;|\Psi^{+}\rangle \\ \sigma_{z}\;\text{for}\;|\Psi^{-}\rangle \end{array}\right.$$ (7)

Now, Bob’s polarization state reads

$$U_{B}\rho_{b}\left(0\right)U_{B}^{†}=[\begin{array}{cc} |\alpha {|}^{2} & {\mathrm{\alpha ;}\beta }^{*}\Lambda_{ab}\left(T_{a},0\right)\\ \alpha^{*}{\beta \Lambda }_{ab}^{*}\left(T_{a},0\right) & |\beta {|}^{2} \end{array}]$$ (8)

It is important to note that, in the above equation, Bob’s qubit’s decoherence function is now exactly the one that previously described the nonlocal coherences for the auxiliary qubit pair. It still has the value Λ*_ab_*(*T_a_*, 0) ≈ 0 (see Eq. 4 and text below that), while Alice’s BSM has remotely entangled Bob’s polarization and frequency. Last, Bob can retrieve the previously hidden information and purify the state 8 by subjecting it to dephasing noise determined by the effective path difference *c*Δ*n_b_T_b_*. With Λ*_ab_*(*T_a_*, *T_b_*) = 1, the final state becomes ∣ϕ⟩. Note that Bob needs no information about Alice’s system-environment correlations.

The biphoton polarization shared by Alice and Bob does not violate the Bell-Clauser-Horne-Shimony-Holt (Bell-CHSH) inequalities during BSM (for details, see the Supplementary Materials). Furthermore, our protocol works even with uncorrelated frequencies, i.e., with *g*(*f_a_*, *f_b_*) = *g_a_*(*f_a_*)*g_b_*(*f_b_*), as opposed to prior teleportation schemes using active nonlocality and fully anticorrelated frequencies to battle decoherence [see, e.g., (*7*)]. Hence, our protocol is not limited to using specific initial polarization states. We did not assume anything about the paths leading to Alice and Bob either; accounting for free evolution, the two time variables in the decoherence function Λ(*t_a_*, *t_b_*) would simply become *t_j_* ↦ *t_j_* + Δ*n*_air_/Δ*n_j_t*_*j*, free_, but, because Δ*n*_air_ ≈ 0, we immediately see that free evolution does not influence our protocol.

Recently, a teleportation protocol starting from a classical two-qubit state was demonstrated in (*36*), where the “hidden nonlocality” of the two auxiliary qubits was first activated with local filters. Our protocol too fits well with the concept of hidden nonlocality (*37*–*39*), albeit our filters are unitaries. However, our protocol shows that nonlocality need not be fully activated before the BSM. As a consequence, the nonlocality of the polarization remains always hidden.

### Experimental results

**Fig. 2.**
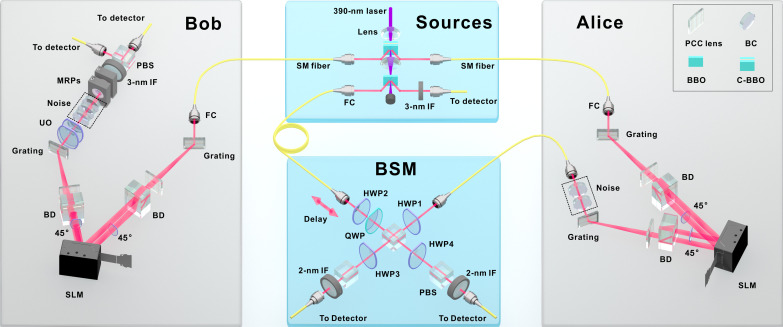
Experimental setup. The setup is composed of four parts. The sources include a polarization entanglement source and a single-photon source. The auxiliary photons’ modulation takes place at “Alice” and “Bob.” After the modulations, the photons from the single-photon source combine in BSM. Noise is implemented in birefringent crystals (BC). C-BBO, sandwich-like BBO + HWP + BBO combination; BBO, beta barium borate; HWP, half-wave plate; QWP, quarter-wave plate; PCC lens, plano-convex cylindrical lens; BD, beam displacer; MRP, motor rotating plate; PBS, polarizing beam splitter; UO, unitary operation; IF, interference filter; SM fiber, single-mode fiber; FC, fiber collimator.

The three photons needed in the protocol were prepared in two consecutive spontaneous parametric down-conversion (SPDC) processes. The phase functions were implemented with SLMs and noise with birefringent crystals. Last, the BSM was carried out in standard manner with linear optical elements. The experimental setup is presented in Fig. 2, and further details are given in the “Experimental design” section.

To thoroughly test our protocol, we teleported the states ∣+⟩ = (∣*H*⟩ + ∣*V*⟩)/√2, ∣−⟩ = (∣*H*⟩ − ∣*V*⟩)/√2, ∣*R*⟩ = (∣*H*⟩ + *i*∣*V*⟩)/√2, and ∣*L*⟩ = (∣*H*⟩ −*i*∣*V*⟩)/√2 using different versions of it. In each case, noise was implemented either on Alice’s side (400λ_0_ of YVO_4_), Bob’s side (411λ_0_ of quartz), or both. In addition, we either used SLMs (i.e., hybrid entanglement) or not. Whenever SLMs were used, Alice’s phase function was θ*_a_*(*f_a_*) = −2π*f_a_*/*c* · 446λ_0_ and Bob’s θ*_b_*(*f_b_*) = −2π*f_b_*/*c* · 429λ_0_. The factors in front of λ_0_ in the phase functions were carefully optimized to mitigate dispersion in the birefringent crystals. This explains the mismatch between the amount of noise and the said factors (400/446 and 411/429).

**Fig. 3.**
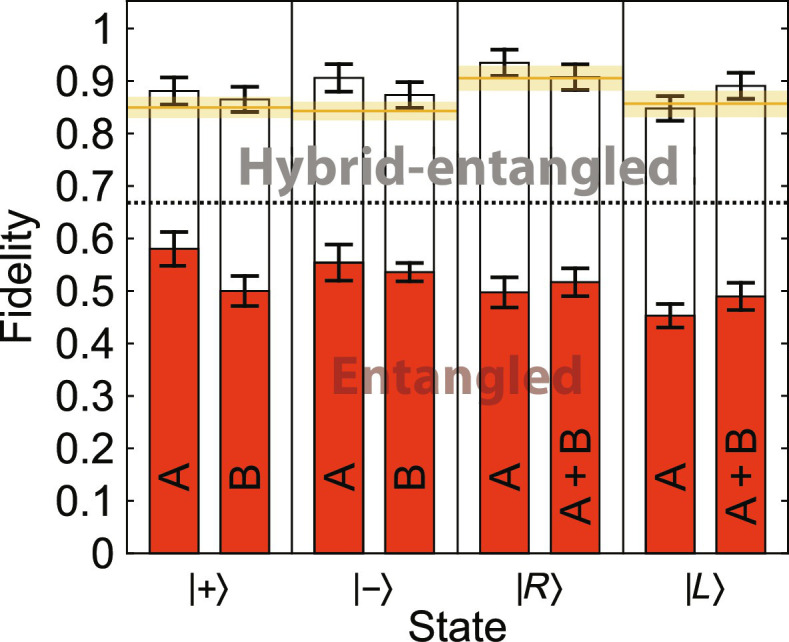
Fidelities of the teleported states. A, noise only on Alice’s side; B, noise only on Bob’s side; A + B, noise on both sides. The lower, red bars correspond to protocols with standard polarization-entangled initial states. The higher, white bars correspond to protocols with hybrid-entangled initial states. Each panel corresponds to different target state, which are given below the panels. The orange lines on top of each panel indicate the reference fidelities with no SLMs and no noise. The black dotted line is the classical average fidelity limit (2/3). The error bars are SDs calculated by a Monte Carlo method and mainly due to the counting statistics.

Figure 3 shows the fidelities of the final states ρ*_f_* with respect to the input states ρ*_i_*, given by $F\left(\rho_{f},\rho_{i}\right)={\left(\text{tr}\sqrt{\sqrt{\rho_{f}}\rho_{i}\sqrt{\rho_{f}}}\right)}^{2}$ . The bars labeled by “A,” “B,” and “A + B” correspond to Alice’s noise configuration, Bob’s noise configuration, and the combination of these two, respectively. The lower, red bars give fidelities of the protocols without SLMs, whereas the higher, white bars give fidelities of the protocols with SLMs. The orange lines correspond to situations, where we had neither noise nor SLMs. The black dotted line is the classical average fidelity limit, 2/3 (*40*).

From Fig. 3, we can clearly see the problem with decoherence even with entangled polarization qubits (lower, red bars) and how hybrid entanglement helps us resolve it (higher, white bars). The white bars labeled by A + B and B describe the main results of this paper and correspond to the protocol starting from Fig. 1 (A and B), respectively. Here, the total state of the two photons before BSM is hybrid-entangled; from the open system’s point of view, the nonlocality is hidden. Still, we achieve very high fidelities in the end, in all cases well above the classical average fidelity limit and approximately equal to the reference fidelities.

The white bars labeled by A correspond to standard teleportation after Alice’s dephasing. Namely, Alice’s dephasing converts the hybrid entanglement into typical polarization entanglement, giving Alice and Bob just the Bell state ∣Ψ^+^⟩ before BSM. Similar results are reported in section S2. There, we purified all the Bell states with SLMs and subsequent dephasing. In addition to YVO_4_ and quartz, we used a 2-m polarization-maintaining single-mode fiber, corresponding better to a real-life scenario.

## DISCUSSION

In this work, we have demonstrated noisy quantum teleportation with the help of multipartite hybrid entanglement. Against the traditional viewpoint of decoherence always acting as a drawback, here dephasing helps us achieve high-fidelity final states. This is due to hybrid entanglement effectively reversing the direction of decoherence. It allows us to start from a classical polarization state and end up with the desired quantum state. Consequently, the resource qubits need not violate the Bell-CHSH inequalities in BSM.

We emphasize that only the initial phase functions need to be controlled. Because neither the frequency distribution nor the frequency correlations play a role in our protocol, we can use any initial Bell state (without coherences) as the auxiliary qubits.

Hybrid entanglement not only helps with fighting decoherence. It can also bring another layer of security. Consider a general setting and Eve the Eavesdropper capturing Bob’s qubit before Bob has purified it with dephasing. Even if Eve knew Alice’s BSM result, she could not purify the captured qubit, because it is not correlated with Eve’s environment. With dephasing, Eve could only make things worse. It would be an interesting line of future research to investigate how deep the teleported information can be hidden, i.e., how large hybrid-entangled total state we can use.

The experimental results presented in the Supplementary Materials suggest that our technique could also be applied in state transfer outside quantum teleportation. In theory, we could transfer any *N*-qubit state across dephasing environments, with a pure state exiting the network. Hence, we could go well beyond decoherence-free subspaces. In practice, only the resolution of the SLMs might limit such applications.

The main assumptions in this work were prior knowledge on the duration of dephasing and access to initial system-environment correlations. Not knowing the lengths of the dephasing channels, Alice and Bob would face an interesting optimization problem in terms of their phase functions. Figure S4 corresponds to such a situation. Should the second assumption fail, it would be worth investigating whether ancillary systems could help, e.g., like Greenberger-Horne-Zeilinger states in (*41*, *42*).

In general, our results highlight the importance of state preparation in the applications of quantum theory and shed new light on entanglement recycling. While our work has a proof-of-principle character, it also opens the possibility to see whether decoherence can be reversed in other physical platforms, including different sources of noise.

## MATERIALS AND METHODS

### Experimental design

First, a combination of beta barium borate (BBO) nonlinear crystal, half-wave plate (HWP), and another BBO (together “C-BBO” in Fig. 2) is pumped by a femtosecond ultraviolet laser (390 nm, 76 MHz). The entangled photon pairs produced by SPDC are distributed to the sides of Alice and Bob.

The photons that did not get down-converted proceed to one more BBO crystal. Here, the state to be teleported is created together with a photon that later triggers the coincidence counting electronics. The state being teleported is finalized by HWP2 and a quarter-wave plate.

Next, in case any noise is to be implemented later, Alice and Bob’s phase functions need to be imprinted on their auxiliary photons. This is achieved by guiding the photons through SLMs that are sandwiched between gratings (1200 liter/mm), plano-convex cylindrical (PCC) lenses, beam displacers (BDs), and HWPs. The gratings and PCC lenses convert the frequency of the photons to spatial degrees of freedom. Because the SLMs are only effective for one polarization component (here for H), the BDs and 45° HWPs are used to change the polarization state into a path state (with H polarization). One hundred fifty SLM pixels cover about 3.5 nm of the photons’ spectra, i.e., their full width at half maximum (FWHM), so that the SLMs can accurately manipulate the phase functions at pixel level. For other pixels, we designed a phase function similar to blazed grating. Its purpose is to diffract excess photons to other angles. The center of the photon spectrum λ_0_ is carefully aligned to the middle of the SLMs.

If the SLMs were used, then the auxiliary photons are now in a multipartite hybrid-entangled state. After state preparation, Alice’s photon is subjected to polarization dephasing in a YVO_4_ crystal so that Alice’s phase function and noise terms cancel each other. If the SLM was used on Bob’s side, then the total state is still hybrid-entangled.

Next, Alice performs BSM on her part of the auxiliary pair and the state being teleported. The BSM entangles Alice’s polarization qubits and remotely transforms the multipartite hybrid entanglement shared by Alice and Bob to local system-environment entanglement on Bob’s side. The BSM is achieved by HWP1, HWP3, HWP4, and three polarizing beam splitters (PBSs). When we measure the Bell states ∣Φ^±^⟩, HWP1 is set to 0°, and HWP3 and HWP4 are set to ±22.5°. For ∣Ψ^±^⟩, HWP1 is set to 45°, and HWP3 and HWP4 are set to ±22.5°. To make the photonic identity better, we use 2-nm-FWHM interference filters in BSM.

Alice communicates her BSM result to Bob. Bob’s unitary operation is composed of two HWPs that change according to Alice’s classical information. Last, after the unitary operation, Bob subjects his photon to polarization dephasing in quartz. Quartz is used in Bob’s setup, while YVO_4_ is used on Alice’s side. This is because YVO_4_ has larger birefringence than quartz, making the thickness of YVO_4_ thinner than that of quartz with the same decoherence and easing the BSM.

Motor rotating plates and a PBS are used to tomograph the photons and receive the teleported information. The gratings and phase functions both reduce the photon counts. The count of fourfold coincidence detections becomes approximately 1/10th, so that the final fourfold coincidence rate is about 0.03 Hz. The data accumulation time for each measurement is 10^4^ s.

## Supplementary Materials

### This PDF file includes:

Sections S1 and S2 Figs. S1 to S4 References
