# Supplementary material for: Overcoming noise in quantum teleportation with multipartite hybrid entanglement
Source: Sci Adv. 2024 May 1;10(18):eadj3435. doi: 10.1126/sciadv.adj3435 (PMC11062577; doi:10.1126/sciadv.adj3435)
Supplement: Supplementary file 1 — Sections S1 and S2 Figs. S1 to S4 References [file sciadv.adj3435_sm.pdf]

Science Advances
AAAS

Supplementary Materials for

Overcoming noise in quantum teleportation with multipartite hybrid entanglement

Zhao-Di Liu *et al.*

Corresponding author: Chuan-Feng Li, cfli@ustc.edu.cn; Jyrki Piilo, jyrki.piilo@utu.fi

Sci. Adv. **10**, eadj3435 (2024)
DOI: 10.1126/sciadv.adj3435

This PDF file includes:

Sections S1 and S2
Figs. S1 to S4
References

Supplementary Materials for

Overcoming noise in quantum teleportation with multipartite hybrid entanglement

Section S1. On hybrid entanglement and hidden nonlocality.

Here, we elaborate on hybrid entanglement and hidden nonlocality. Let us first show that, following the state preparation, we can go arbitrarily close to zero with the degree of entanglement in polarization, i.e., the degree of freedom being teleported. The bipartite polarization state in question reads

$$\rho_{ab}(t_a,t_b)=\frac{1}{2}\begin{pmatrix}0 & 0 & 0 & 0\\ 0 & 1 & \Lambda_{ab}(t_a,t_b) & 0\\ 0 & \Lambda_{ab}^*(t_a,t_b) & 1 & 0\\ 0 & 0 & 0 & 0\end{pmatrix}, \tag{S1}$$

where $\Lambda(t_a,t_b)=\int df_a df_b |g(f_a,f_b)|^2 e^{i[2\pi f_a \Delta n_a(t_a-T_a)-2\pi f_b \Delta n_b(t_b-T_b)]}$, t_j are the interaction times, and $-T_j$ are the slopes of the linear phase functions, fixed in our experimental setups with spatial light modulators (SLMs).

To quantify entanglement in ρ_{ab}, we use the standard definition of concurrence in two-qubit systems, i.e., $C(\rho_{ab})=\max\{0,\lambda_1-\lambda_2-\lambda_3-\lambda_4\}$, where λ_j are decreasingly ordered eigenvalues of the matrix $\sqrt{\sqrt{\rho_{ab}}(\sigma_y\otimes\sigma_y)\rho_{ab}^*(\sigma_y\otimes\sigma_y)\sqrt{\rho_{ab}}}$ (*43*). In our case, one obtains $C(\rho_{ab}(t_a,t_b))=|\Lambda_{ab}(t_a,t_b)|$. Because we are interested in noise appearing on Bob's side *after* Alice's Bell-state measurement (BSM), we may set $t_b=0$. By also assuming that $T_j\gg 0$, we get rapidly oscillating phase factors that cancel each other, yielding $|\Lambda_{ab}(t_a,0)|\approx 0$. Hence, $C(\rho_{ab}(t_a,0))\approx 0$ with all interaction times t_a, even $t_a=T_a$. If we had $t_j=T_j$ with $j=b$ as well, it would follow from the normalization condition of the frequency spectrum that $|\Lambda_{ab}(T_a,T_b)|=1$ and thus $C(\rho_{ab}(T_a,T_b))=1$. That is, maximum entanglement would emerge from local "filtering", which is the loose definition of hidden nonlocality (*36*–*39*). However, the nonlocality of the open system remains hidden in our case, meaning that any violation of the Bell-CHSH inequalities (*1*) cannot be experimentally detected during BSM in the bipartite polarization's Hilbert space.

Examining the total system consisting of both the open system and its environment reveals "where" the former's nonlocality is actually hidden. Namely, the total auxiliary system shared by Alice and Bob reads

$$|\Psi(t_a,0)\rangle=\frac{1}{\sqrt{2}}\{|HV\rangle\int df_a df_b g(f_a,f_b)e^{i[\theta_{aH}(f_a)+2\pi f_a n_{aH} t_a+\theta_{bV}(f_b)]}|f_a f_b\rangle$$
$$+|VH\rangle\int df_a df_b g(f_a,f_b)e^{i[\theta_{aV}(f_a)+2\pi f_a n_{aV} t_a+\theta_{bH}(f_b)]}|f_a f_b\rangle\}. \tag{S2}$$

Clearly $\langle HV|VH\rangle=0$, but also $\int df_a' df_b' g^*(f_a',f_b')e^{-i[\theta_{aH}(f_a')+2\pi f_a' n_{aH} t_a+\theta_{bV}(f_b')]}\langle f_a' f_b'|$

$\times \int df_a df_b g(f_a, f_b) e^{i[\theta_{aV}(f_a)+2\pi f_a n_{aV} t_a+\theta_{bH}(f_b)]} |f_a f_b\rangle = \Lambda^*_{ab}(t_a, 0) \approx 0$ with $T_j \gg 0$. Therefore, because $|\Psi(t_a, 0)\rangle$ is a balanced superposition of two pairwise orthogonal states, it is fully entangled, and because the states represent different degrees of freedom, one can talk about (multipartite) hybrid entanglement (*33–35*).

Above, we saw that the phase functions determine the degree of hybrid entanglement between the composite open system and the composite environment. Alternatively, one could investigate entanglement between Alice and Bob's subsystems consisting of polarization and frequency. Because both the initial phase functions and the subsequent dephasing can be modeled with local unitaries, the purity of Alice and Bob's subsystems remains constant, and so does the entanglement between them. In this case, concurrence is defined by $C(\Psi_{\mathrm{AB}}) = \sqrt{2(1 - \mathrm{tr}[\sigma_A^2])} = \sqrt{2(1 - \mathrm{tr}[\sigma_B^2])}$, where $\sigma_{A(B)}$ is Alice's (Bob's) polarization-frequency state (*44*). Consequently, while the phase functions determine the degree of $\mathcal{S}$–$\mathcal{E}$ entanglement, the initial probability amplitudes determine the degree of $\mathcal{A}$–$\mathcal{B}$ entanglement. Interestingly, it follows that Alice and Bob's photons can be hybrid-entangled without there being any polarization-polarization or frequency-frequency entanglement. It should be mentioned that classical polarization-polarization correlations are still needed for our teleportation protocol to work.

Supplementary Materials for

Overcoming noise in quantum teleportation with multipartite hybrid entanglement

Section S2. Experimental verification of state purification with "reverse decoherence".

To test state purification with "reverse decoherence" in a clearer fashion, we purified the entire Bell basis $\{|\Phi^{\pm}\rangle, |\Psi^{\pm}\rangle\}$ with different noise configurations. Although these results better illustrate the effect of the phase functions $\theta_j(f_j)$ on the decoherence dynamics, it is important to notice that such purification is *not* needed for our teleportation protocol to work. The experimental setup, which can be obtained from the teleportation setup by simply blocking the pump laser before the BBO, inserting QWP1 on Alice's path, and setting HWP3 and HWP4 to 0°, is shown in Fig. S1.

First, we placed an yttrium orthovanadate (YVO_4) plate on Alice's path, matching with the effective path difference $c\Delta n_a T_a = 400\lambda_0$ (λ_0 = 780 nm). We constructed the phase function $\theta_a(f_a)$ with an SLM preceding the YVO_4 plate and changed its slope gradually to cancel the path difference and reach maximum purity. Secondly, we fixed Bob's phase function as $\theta_b(f_b) = -2\pi f_b/c \cdot 429\lambda_0$ and simulated subsequent dephasing by stacking multiple quartz plates on his path.

The resulting fidelities of both configurations are plotted in Fig. S2 with the target Bell states shown in the upper left corners. Note that, in Alice's case, the horizontal axes give the factor x in $\theta_a(f_a) = -2\pi f_a/c \cdot x$ and not the thickness of YVO_4. In all cases, the bipartite polarization starts as mixed and ends as pure at approximately $450\lambda_0$ for Alice and $400\lambda_0$ for Bob. The experimental data is in good agreement with the theoretical predictions, which were evaluated numerically by using the actual SLM-pixel values, fitted frequency spectra (see Fig. S3), and Sellmeier equations of the birefringent media.

We suspect that the theory and experiment are in better agreement on Alice's side for two reasons: First, we used only one YVO_4 plate in the experiment, which means that small angles between other plates could not affect the results. Secondly, here we imprinted the phase function $\theta_a(f_a)$ on relatively wider frequency domain than on Bob's side, where a wider interference filter (3 nm) was used. That is, more photons *without* the phase function $\theta_b(f_b)$ got through.

Finally, to simulate a more realistic scenario, we fixed the amount of dephasing with a 2 m polarization maintaining single-mode fiber (PM fiber) on Bob's side and purified the state $|\Psi^+\rangle$ by changing the slope of $\theta_b(f_b)$ (see Fig. S4A). We obtained the highest purity with $\theta_b(f_b) = -2\pi f_b/c \cdot 1063\lambda_0$. The amount of noise, $1080\lambda_0$, was used as a fit parameter. The exact amount of noise was not known due to the manufacturer of the fiber not reporting the fiber's Sellmeier equation. Hence, dispersion was not accounted for, which explains the difference between the maxima of theory and experiment. To further visualize our protocol, we have plotted the real values of the initial and time-evolved density matrix elements in Figs. S4B and S4C, respectively. The imaginary values were negligibly small.

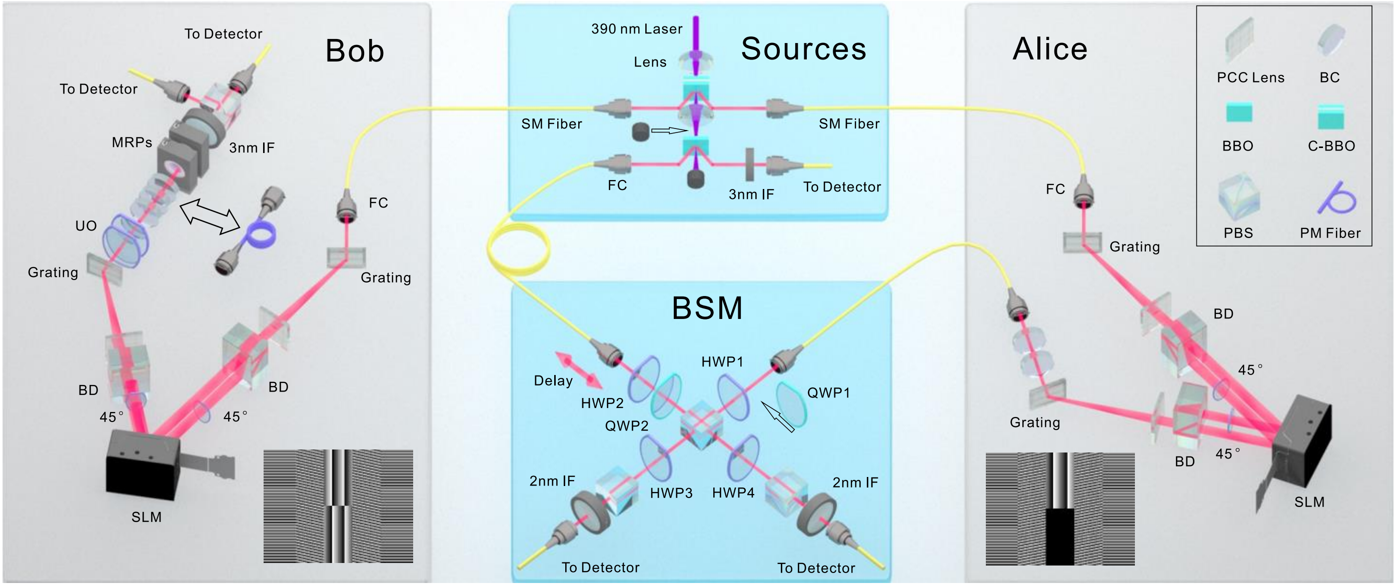

Fig. S1. Experimental setup of state purification. The setup can be changed from noisy quantum teleportation to state purification by blocking the 390 nm pump laser between C-BBO (sandwich-like BBO+HWP+BBO combination) and BBO (beta barium borate) and inserting QWP1 (quarter-wave plate) in the setup. Here, we also use a PM fiber (polarization maintaining single-mode fiber) on Bob's side, corresponding to "real-life noise". Alice and Bob's SLMs are accompanied by sample holograms in the picture. The sample hologram on Alice's side matches with $400\lambda_0$ of YVO_4, while the sample hologram on Bob's side matches with 2 m polarization maintaining single-mode fiber (PM fiber). HWP—half-wave plate, PCC lens—plano-convex cylindrical lens, BD—beam displacer, BC—birefringent crystal, MRP—motor rotating plate, PBS— polarizing beam splitter, UO—unitary operation, IF—interference filter, SM fiber—single-mode fiber, FC—fiber collimator.

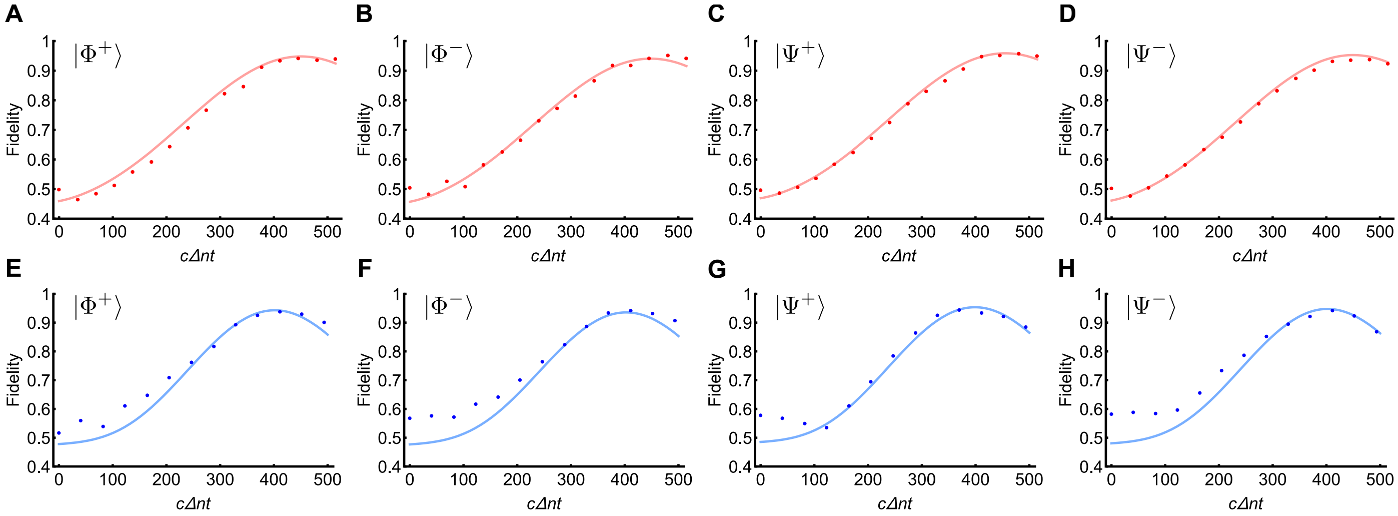

Fig. S2. Results of state purification with "reverse decoherence". Fidelities of the purified states as functions of the effective path difference in units of λ_0. The target Bell states are shown in the upper left corners. The solid curves represent the theoretical predictions, while the dots correspond to the measurement data. The error bars are standard deviations calculated by a Monte Carlo method and of the same size as the dots. The numerical values of the largest error bars in each of the panels are: (**A**) 0.0040, (**B**) 0.0039, (**C**) 0.0055, (**D**) 0.0040, (**E**) 0.0048, (**F**) 0.0044, (**G**) 0.0035, and (**H**) 0.0046.

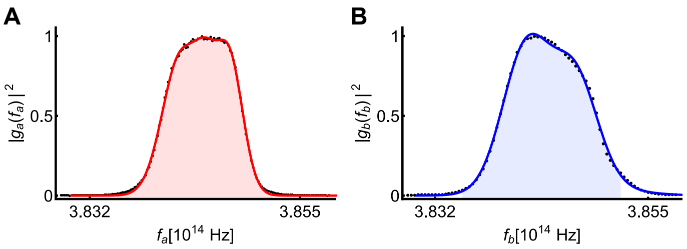

Fig. S3. The frequency spectra. (A) Alice's margin of the joint frequency spectrum after the 2 nm interference filter. **(B)** Bob's margin of the joint frequency spectrum after the 3 nm interference filter. The black dots are the measured (and scaled) counts. The solid curves are the fitted functions, i.e., the weighted sums of three Gaussians. The filled areas represent the frequency domains of the phase functions**.**

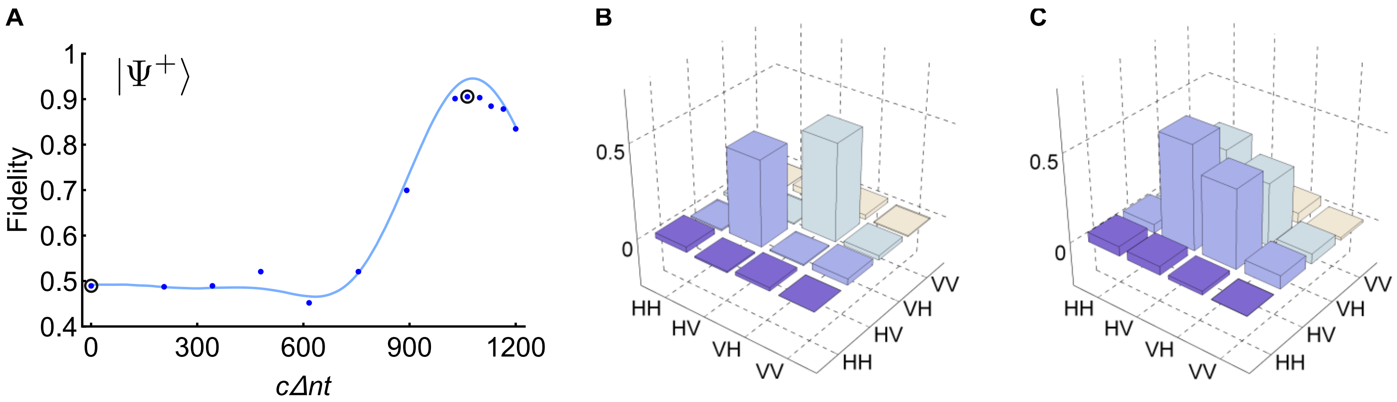

Fig. S4. State purification in an optical fiber. (A) Fidelity of the purified state $|\Psi^+\rangle$ as a function of the effective path difference in units of λ_0. The solid curve represents the theoretical prediction, while the dots correspond to the measurement data. The error bars are standard deviations calculated by a Monte Carlo method and of the same size or smaller as the dots. The numerical value of the largest error bar is 0.0049. **(B)**, **(C)** Real values of **(B)** the initial and **(C)** time-evolved density matrix elements, corresponding to the circled data points in panel **(A)**.

REFERENCES AND NOTES

1. M. A. Nielsen, I. L. Chuang, *Quantum Computation and Quantum Information* (Cambridge University Press, Cambridge, 2000).

2. C. H. Bennett, G. Brassard, C. Crépeau, R. Jozsa, A. Peres, W. K. Wootters, Teleporting an unknown quantum state via dual classical and Einstein-Podolsky-Rosen channels. *Phys. Rev. Lett.* **70**, 1895–1899 (1993).

3. E.-M. Laine, H.-P. Breuer, J. Piilo, Nonlocal memory effects allow perfect teleportation with mixed states. *Sci. Rep.* **4**, 4620 (2014).

4. X.-M. Hu, C. Zhang, C.-J. Zhang, B.-H. Liu, Y.-F. Huang, Y.-J. Han, C.-F. Li, G.-C. Guo, Experimental certification for nonclassical teleportation. *Quantum Eng.* **1**, e13 (2019).

5. K. Sun, Y. Wang, Z.-H. Liu, X.-Y. Xu, J.-S. Xu, C.-F. Li, G.-C. Guo, A. Castellini, F. Nosrati, G. Compagno, R. Lo Franco, Experimental quantum entanglement and teleportation by tuning remote spatial indistinguishability of independent photons. *Opt. Lett.* **45**, 6410–6413 (2020).

6. X.-M. Hu, C. Zhang, B.-H. Liu, Y. Cai, X.-J. Ye, Y. Guo, W.-B. Xing, C.-X. Huang, Y.-F. Huang, C.-F. Li, G.-C. Guo, Experimental high-dimensional quantum teleportation. *Phys. Rev. Lett.* **125**, 230501 (2020).

7. Z.-D. Liu, Y.-N. Sun, B.-H. Liu, C.-F. Li, G.-C. Guo, S. Hamedani Raja, H. Lyyra, J. Piilo, Experimental realization of high-fidelity teleportation via a non-Markovian open quantum system. *Phys. Rev. A* **102**, 062208 (2020).

8. C. H. Bennett, S. J. Wiesner, Communication via one- and two-particle operators on Einstein-Podolsky-Rosen states. *Phys. Rev. Lett.* **69**, 2881–2884 (1992).

9. B.-H. Liu, X.-M. Hu, Y.-F. Huang, C.-F. Li, G.-C. Guo, A. Karlsson, E.-M. Laine, S. Maniscalco, C. Macchiavello, J. Piilo, Efficient superdense coding in the presence of non-Markovian noise. *Europhys. Lett.* **114**, 10005 (2016).

10. A. Karlsson, H. Lyyra, E.-M. Laine, S. Maniscalco, J. Piilo, Non-Markovian dynamics in two-qubit dephasing channels with an application to superdense coding. *Phys. Rev. A* **93**, 032135 (2016).

11. P. W. Shor, J. Preskill, Simple proof of security of the BB84 quantum key distribution protocol. *Phys. Rev. Lett.* **85**, 441–444 (2000).

12. C. Lee, Z. Zhang, G. R. Steinbrecher, H. Zhou, J. Mower, T. Zhong, L. Wang, X. Hu, R. D. Horansky, V. B. Verma, A. E. Lita, R. P. Mirin, F. Marsili, M. D. Shaw, S. W. Nam, G. W. Wornell, F. N. C. Wong, J. H. Shapiro, D. Englund, Entanglement-based quantum communication secured by nonlocal dispersion cancellation. *Phys. Rev. A* **90**, 062331 (2014).

13. S. Utagi, R. Srikanth, S. Banerjee, Ping-pong quantum key distribution with trusted noise: Non-Markovian advantage. *Quantum Inf. Process.* **19**, 366 (2020).

14. H.-P. Breuer, F. Petruccione, *The Theory of Open Quantum Systems* (Oxford University Press, 2007).

15. K. Sinha, P. Meystre, E. A. Goldschmidt, F. K. Fatemi, S. L. Rolston, P. Solano, Non-Markovian collective emission from macroscopically separated emitters. *Phys. Rev. Lett.* **124**, 043603 (2020).

16. A. Carmele, N. Nemet, V. Canela, S. Parkins, Pronounced non-Markovian features in multiply excited, multiple emitter waveguide QED: Retardation induced anomalous population trapping. *Phys. Rev. Res.* **2**, 013238 (2020).

17. S. Lorenzo, S. Longhi, A. Cabot, R. Zambrini, G. L. Giorgi, Intermittent decoherence blockade in a chiral ring environment. *Sci. Rep.* **11**, 12834 (2021).

18. Y. Maleki, B. Ahansaz, Maximal-steered-coherence protection by quantum reservoir engineering. *Phys. Rev. A* **102**, 020402(R) (2020).

19. R. Stárek, M. Mičuda, I. Straka, M. Nováková, M. Dušek, M. Ježek, J. Fiurášek, R. Filip, Experimental quantum decoherence control by dark states of the environment. *New J. Phys.* **22**, 093058 (2020).

20. W. Wu, Z.-Z. Zhang, Controllable dynamics of a dissipative two-level system. *Sci. Rep.* **11**, 7188 (2021).

21. Y. Peng, H. Fan, Achieving the Heisenberg limit under general Markovian noise using quantum error correction without ancilla. *Quantum Inf. Process.* **19**, 266 (2020).

22. A. E. Seedhouse, T. Tanttu, R. C. C. Leon, R. Zhao, K. Y. Tan, B. Hensen, F. E. Hudson, K. M. Itoh, J. Yoneda, C. H. Yang, A. Morello, A. Laucht, S. N. Coppersmith, A. Saraiva, A. S. Dzurak, Pauli blockade in silicon quantum dots with spin-orbit control. *PRX Quantum* **2**, 010303 (2021).

23. P. Parrado-Rodríguez, C. Ryan-Anderson, A. Bermudez, M. Müller, Crosstalk suppression for fault-tolerant quantum error correction with trapped ions. *Quantum* **5**, 487 (2021).

24. Y. Dong, Y. Zheng, S. Li, C.-C. Li, X.-D. Chen, G.-C. Guo, F.-W. Sun, Non-Markovianity-assisted high-fidelity Deutsch–Jozsa algorithm in diamond. *Npj Quantum Inf.* **4**, 3 (2018).

25. Q. Yao, J. Zhang, X.-F. Yi, L. You, W. Zhang, Uniaxial dynamical decoupling for an open quantum system. *Phys. Rev. Lett.* **122**, 010408 (2019).

26. A. M. Souza, Process tomography of robust dynamical decoupling with superconducting qubits, *Quantum Inf. Process.* **20**, 237 (2021).

27. P. G. Kwiat, A. J. Berglund, J. B. Altepeter, A. G. White, Experimental verification of decoherence-free subspaces. *Science* **290**, 498–501 (2000).

28. J. B. Altepeter, P. G. Hadley, S. M. Wendelken, A. J. Berglund, P. G. Kwiat, Experimental investigation of a two-qubit decoherence-free subspace. *Phys. Rev. Lett.* **92**, 147901 (2004).

29. B.-H. Liu, L. Li, Y.-F. Huang, C.-F. Li, G.-C. Guo, E.-M. Laine, H.-P. Breuer, J. Piilo, Experimental control of the transition from Markovian to non-Markovian dynamics of open quantum systems. *Nat. Phys.* **7**, 931–934 (2011).

30. E.-M. Laine, H.-P. Breuer, J. Piilo, C.-F. Li, G.-C. Guo, Nonlocal memory effects in the dynamics of open quantum systems. *Phys. Rev. Lett.* **108**, 210402 (2012).

31. B.-H. Liu, D.-Y. Cao, Y.-F. Huang, C.-F. Li, G.-C. Guo, E.-M. Laine, H.-P. Breuer, J. Piilo, Photonic realization of nonlocal memory effects and non-Markovian quantum probes. *Sci. Rep.* **3**, 1781 (2013).

32. Z.-D. Liu, H. Lyyra, Y.-N. Sun, B.-H. Liu, C.-F. Li, G.-C. Guo, S. Maniscalco, J. Piilo, Experimental implementation of fully controlled dephasing dynamics and synthetic spectral densities. *Nat. Commun.* **9**, 3453 (2018).

33. P. van Loock, Optical hybrid approaches to quantum information. *Laser Photon. Rev.* **5**, 167–200 (2011).

34. K. Huang, H. Le Jeannic, O. Morin, T. Darras, G. Guccione, A. Cavaillès, J. Laurat, Engineering optical hybrid entanglement between discrete- and continuous-variable states. *New J. Phys.* **21**, 083033 (2019).

35. S. Takeda, T. Mizuta, M. Fuwa, P. van Loock, A. Furusawa, Deterministic quantum teleportation of photonic quantum bits by a hybrid technique. *Nature* **500**, 315–318 (2013).

36. J.-Y. Li, X.-X. Fang, T. Zhang, G. N. M. Tabia, H. Lu, Y.-C. Liang, Activating hidden teleportation power: Theory and experiment. *Phys. Rev. Res.* **3**, 023045 (2021).

37. S. Popescu, Bell's inequalities and density matrices: Revealing "hidden" nonlocality. *Phys. Rev. Lett.* **74**, 2619–2622 (1995).

38. N. Gisin, Hidden quantum nonlocality revealed by local filters. *Phys. Lett. A* **210**, 151–156 (1996).

39. P. G. Kwiat, S. Barraza-Lopez, A. Stefanov, N. Gisin, Experimental entanglement distillation and ‘hidden’ non-locality. *Nature* **409**, 1014–1017 (2001).

40. S. Massar, S. Popescu, Optimal extraction of information from finite quantum ensembles. *Phys. Rev. Lett.* **74**, 1259–1263 (1995).

41. A. Karlsson, M. Bourennane, Quantum teleportation using three-particle entanglement. *Phys. Rev. A* **58**, 4394–4400 (1998).

42. A. Barasiński, A. Černoch, K. Lemr, Demonstration of controlled quantum teleportation for discrete variables on linear optical devices. *Phys. Rev. Lett.* **122**, 170501 (2019).

43. W. K. Wootters, Entanglement of formation of an arbitrary state of two qubits, *Phys. Rev. Lett.* **80**, 2245–2248 (1998).

44. P. Rungta, V. Bužek, C. M. Caves, M. Hillery, G. J. Milburn, Universal state inversion and concurrence in arbitrary dimensions, *Phys. Rev. A* **64**, 042315 (2001).
